# Study of the Impact of Epoxidized Soybean Oil on the Characteristics of Wood-Polymer Composites

**DOI:** 10.3390/ma18112455

**Published:** 2025-05-23

**Authors:** Andrii Kulikov, Dmytro Kryvolapov, Kostyantyn Sukhyy, Oleksandr Yeromin, Marcel Fedak, Olena Prokopenko, Iryna Sukha, Angelo Musaio, Tomas Hrebik

**Affiliations:** 1 Faculty of Manufacturing Technologies with the Seat in Presov, Technical University of Kosice, Bayerova 1, 08001 Presov, Slovakia; marcel.fedak@tuke.sk (M.F.); tomas.hrebik@tuke.sk (T.H.); 2 Department of Ecology, Heat Transfer and Labor Protection, Faculty of Mechanical Engineering and Environmental Protection, Ukrainian State University of Science and Technologies, 49000 Dnipro, Ukraine; dmipro706@gmail.com (D.K.); ksukhyy@gmail.com (K.S.); aoeremin@gmail.com (O.Y.); eprok777@ukr.net (O.P.); irinasuha3@gmail.com (I.S.); 3 Rectorate, Università degli Studi di Genova, 16167 Genova, Italy; angelo.musaio@unige.it

**Keywords:** water absorption, wood-polymer composite, epoxidized soybean oil, high-density polyethylene, physical and mechanical properties

## Abstract

The effect of epoxidized soybean oil on the operational, technological, and physical and mechanical properties of composites based on high-density recycled polyethylene filled with wood floor was investigated. It has been shown that the introduction of epoxidized soybean oil in the amount of 0.5 wt.% into the wood-polymer composite (WPC) improves the physical, mechanical, and operational properties of the material: the Charpy impact strength (on notched samples) increases from 7.5 kJ/m^2^ to 20.0 kJ/m^2^, the bending strength increases from 31.6 MPa to 50.8 MPa, and the coefficient of linear thermal expansion decreases by 15%. With a further increase in the content of epoxidized soybean oil in the composite, its water absorption and technological shrinkage decrease, but its physical and mechanical properties deteriorate.

## 1. Introduction

Soybean is an industrial crop primarily grown for soybean oil and protein production, ranking among the highest-yielding oilseed crops worldwide. Today, economically viable industrial applications of soybean oil include paper coatings, alkyd resins, and oleochemical products such as epoxidized soybean oil. Vegetable oils can be modified by converting double bonds into more reactive groups, such as epoxides, acrylates, and hydroxyls. Among these reactions, epoxidation is preferred due to the high reactivity of the oxirane ring and the wide range of potential applications for the resulting products [1]. Given the focus on energy savings and environmental concerns, it is essential to develop low-carbon, eco-friendly, renewable biomass-based materials to replace petrochemical materials, particularly in the construction industry and building materials sector. In recent years, biological materials have gained popularity in material production due to their cost competitiveness, renewability, and abundant availability—especially for manufacturing wood adhesives. Additionally, research has been conducted on bio-based construction materials, such as composites made by mixing straw and lime paste as an aggregate, hempcrete, and composite materials from hemp flax. These bio-based composite materials have demonstrated acceptable mechanical properties, making them suitable for use in structural and construction applications. Natural vegetable oils, particularly soybean oil, have high practical value due to their fatty acid and triglyceride content exceeding 99%. Utilizing discarded or recycled soybean oil in the production of building materials represents an environmentally friendly and sustainable approach. The synthesis of epoxidized soybean oil (ESO) through an epoxidation reaction enhances the reactivity of soybean oil. ESO serves as an eco-friendly cross-linking agent, containing multiple epoxide groups within its molecules, allowing it to rapidly cross-link with reactants to improve the strength and water resistance of materials. Bio-based resins derived from epoxidized soybean oil have been successfully applied in the production of grafted fibers, coatings, adhesives, and multifunctional composite materials. Therefore, the use of ESO-based composite materials as a substitute for petrochemical-based materials can contribute to energy savings and environmental protection [2].

The development of bio-based thermoset materials requires selecting an oil with an unsaturated structure that contains highly reactive functional groups, such as carbon-carbon double bonds. Due to its natural appeal, the advancement of bio-based materials has garnered significant societal interest, with soybean oil derivatives emerging as a focal point. In traditional wood–plastic composites, more expensive coupling agents are often used, such as maleinized polyolefins. In contrast, epoxidized soybean oil can serve as a cost-effective alternative, helping to reduce overall production costs while maintaining the material’s performance characteristics. Among them, epoxidized soybean oil (ESO) stands out as the most extensively studied raw material for synthesizing epoxy resins (EPs). The preference for it is due to its high content of unsaturated fatty acids, which allow the efficient incorporation of epoxy groups, as well as its biodegradability and renewable nature, making it an environmentally friendly alternative to petroleum-based precursors. Additionally, ESO exhibits good compatibility with various curing agents, enabling the formulation of epoxy resins with desirable mechanical and thermal properties. Its relatively low cost compared to conventional epoxy precursors further enhances its appeal for sustainable material development. An alternative approach involves subjecting ESO to an acrylation process, resulting in acrylated epoxidized soybean oil (AESO). While this material forms a thermoset through radical polymerization, it exhibits suboptimal mechanical properties and low glass transition temperatures [3]. Epoxidized soybean oil (ESO) contains multiple epoxide groups in its molecular structure, allowing it to effectively cross-link with reactants, thereby enhancing the mechanical strength and water resistance of polymers. ESO is considered an environmentally friendly material [4]. Wood-polymer composites are thermoplastic polymers containing a certain amount of wood in the form of flour or short fibers [5]. These materials can be easily processed using typical woodworking procedures and can also be processed into products by extrusion or injection molding [6]. They are typically used as a wood substitute (e.g., for fencing, flooring, and decking). Their use is convenient in humid environments or in contact with water: the hydrophobic polymer insulates and protects hydrophilic wood fibers, which increases the durability and reduces maintenance interventions [7]. A WPC is also used in acoustics [8] and in the automotive industry [9].

A WPC is used mainly for exterior work. In particular, this material is used to make terrace floors, siding, decorative fences, fence systems, steps, universal profiles, various accessories and components for exterior decoration. An important limitation of the polymers used in WPCs is the requirements for processing conditions (temperature, pressure) that do not lead to the thermal decomposition of the wood filler. Wood decomposes at a temperature of about 220 °C, so general-purpose polymers are commonly used for WPC production: polyethylene [10], polypropylene [11], polyvinyl chloride [12], and polystyrene [13]. The main processes for processing wood-polymer composites are extrusion, injection molding, and compression molding or thermoforming (pressing). New manufacturing processes for WPCs include additive manufacturing using fused layer modeling and laser sintering [14,15,16,17]. As for the properties of finished products, each method has its own set of parameters, advantages, and disadvantages [18,19]. The main advantages of wood as a filler are the reduced costs and improved environmental performance of the resulting composite. The former is quite obvious, since wood particles are usually inexpensive materials that are often obtained from wood or industrial waste. The latter is also obvious, since the corresponding fraction, sometimes reaching 60 or 70 wt.%, of non-biodegradable material of fossil origin is replaced by an environmentally friendly component [20,21]. But natural fibers as fillers have their drawbacks. Wood fibers are hydrophilic due to the hydroxyl groups contained in the molecular chains of cellulose and hemicellulose. And most thermoplastics used for WPCs are hydrophobic. Wood fibers agglomerate during compounding due to physical (hydrogen) bonds. As a result, wood can be poorly dispersed in polymer matrices, and thus its reinforcing effect can be impaired. The mechanical properties of composites can decrease when the aggregation of filler particles occurs [6,21]. To improve the adhesion at the polymer–wood filler interface, aprotectants are used, the functional groups of which interact with the surface functional groups of the wood filler [22]. Many apretizing agents have been used in WPCs: silicates, titanates, organic acids, chlorotriazines, acid anhydrides, isocyanates, acrylates, amides, imides, silanes, polymeric compounds, etc. [23,24].

The aim of this study is to investigate the effect of using epoxidized soybean oil as a binder modifier on the physical, mechanical, and operational properties of a WPC based on recycled high-density polyethylene. This additive was selected due to its high reactivity, which allows it to effectively interact with the polymer matrix and improve adhesion between components. Additionally, ESO is widely available and cost-effective, making it a practical alternative to other bio-based oils. Its biodegradability and renewable nature align with sustainability goals. Moreover, ESO was used to investigate its properties as an organic material with the aim of reducing the CO_2_ footprint of the WPC.

## 2. Materials and Methods

A composite with the following composition was used as the object of study [25]:

- High-density recycled polyethylene—30 wt.%;
- Wood floor—54 wt.%;
- Calcite—11 wt.%;
- Polyethylene wax—1.1 wt.%;
- Stearic acid—1.1 wt.%;
- UV stabilizer and colorants.

The content of epoxidized soybean oil (epoxy number 6.6%) in the composition was varied from 0 to 3 wt.%. This range was selected based on preliminary considerations regarding ESO’s compatibility with the polymer matrix and its potential effects on composite properties. Higher concentrations could lead to phase separation, negatively affecting mechanical integrity and adhesion within the composite. Additionally, previous studies on bio-based modifiers suggest that beyond a certain threshold, further increases in concentration do not yield proportional improvements and may even compromise structural stability. Thus, the chosen range allows for a systematic evaluation of ESO’s influence while maintaining processability and cost-effectiveness in the development of WPC composition.

The physical and mechanical properties of the used recycled high-density polyethylene are given in Table 1.

It should be noted that the used recycled HDPE is first recycled, meaning it has undergone only one recycling cycle. Its properties remain comparable to those of virgin HDPE, while offering a lower cost and greater environmental benefits.

In the current work, oak wood flour of the M200 brand according to DEST 16361-87 was used. The main characteristics of wood flour of the M200 brand are given in Table 2.

As a mineral filler, untreated microcalcite of the MMC-1 brand produced by ChAT “Novgorod-Seversky Building Materials Plant”, Ukraine, was used. As lubricants, stearic acid of the 1860 brand produced by PT. Dua Kuda, Indonesia, and polyethylene wax of the CWN-105 brand produced by Liebenzell AG Group Company, Valencia, Spain were used.

The main stages of preparation of samples of wood-polymer composite included the following stages: preparation of raw materials, weighing of components, mixing, granulation, extrusion of terrace board, cooling and cutting, and finishing.

**Figure 1:**
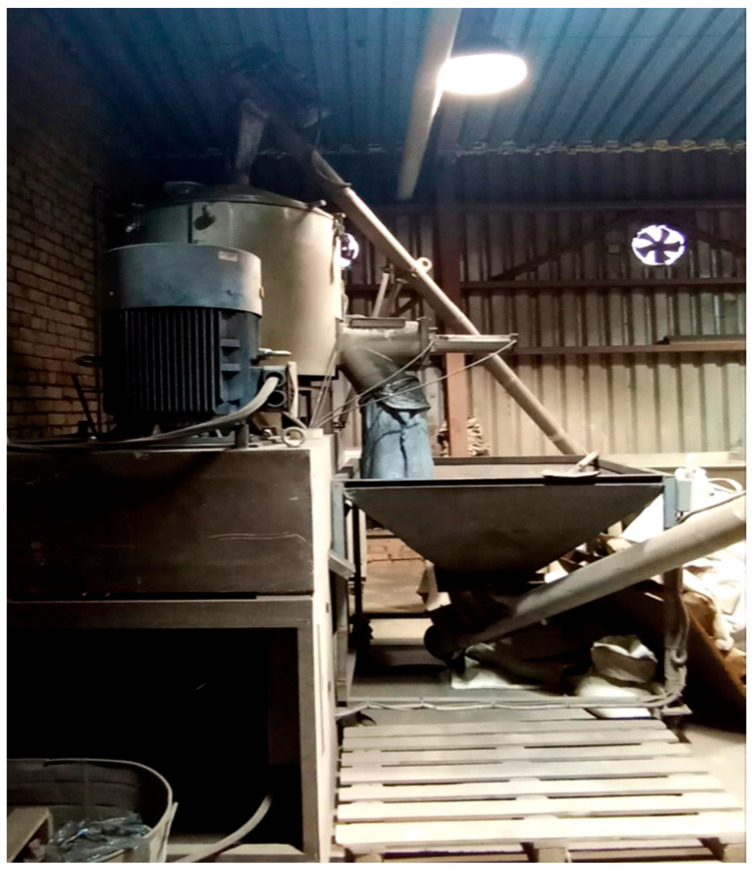
Paddle mixer SKY WIN SWHL 600/1200.

The preparatory stage includes the quality control of raw materials and the preliminary drying of components (if necessary). Quality control involved the visual inspection of the raw materials to ensure uniform granule size, the absence of contaminants, and consistent coloration. Additionally, for recycled HDPE, key parameters such as density and the melt flow index were verified against supplier specifications to confirm compliance with expected material characteristics. Then, on electronic scales (3rd accuracy class), the components were weighed and dosed into appropriate containers with a total mass of 150 kg. After that, using a screw feeder, the raw material was fed into a blade mixer model skywinswhl 600/1200 (Figure 1), where at a blade speed of 800 rpm, a mechanical mixture was prepared for 45–60 min until a temperature of 100 °C was reached to ensure the removal of residual moisture. Epoxidized soybean oil (ESO) was added last during the loading of components into the mixer. This ensured its uniform distribution throughout the polymer–wood flour mixture. The controlled mixing time and temperature facilitated the effective dispersion of ESO, optimizing its role as a binder modifier before further processing.

**Figure 2:**
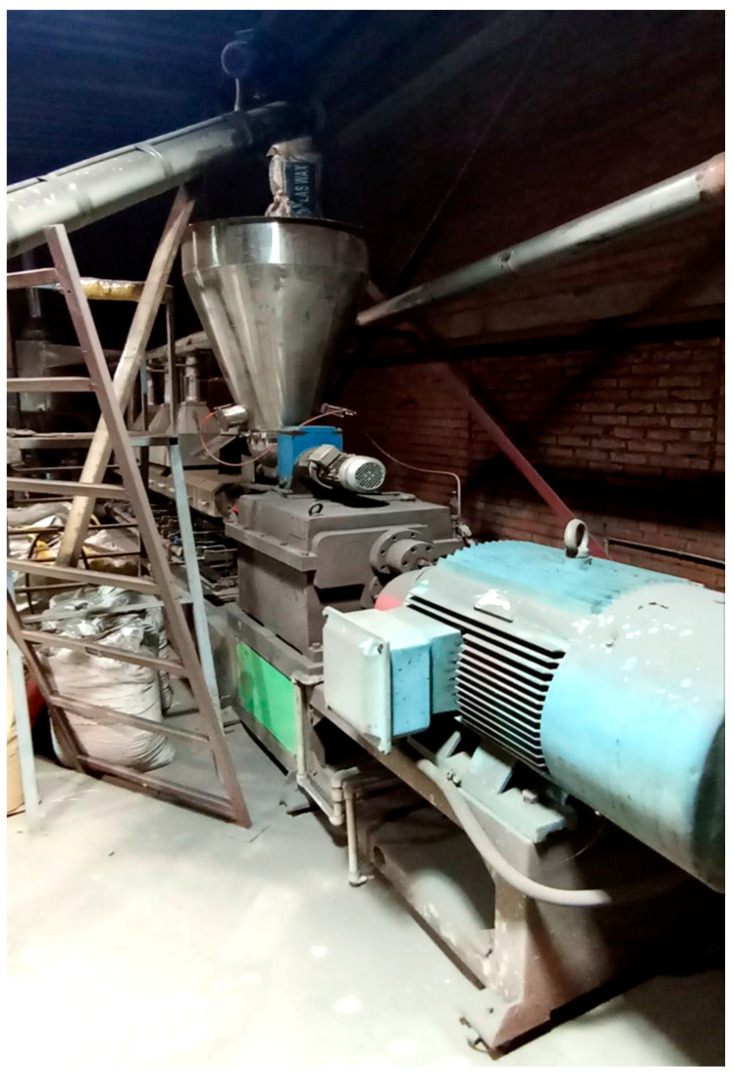
Twin-screw extruder SKY WIN SWMSZ-3.

Next, the mixture was fed into a twin-screw granulator of the skywin swmsz-3 brand (Figure 2), where the composite material was homogenized at a temperature of 180 °C and screw speeds of 250–350 rpm. The composite material in the form of granules was loaded into big bags and transported to the decking board extrusion line.

Extrusion of the terrace board took place on a twin-screw extruder manufactured by KraussMaffei (Munich, Germany), model KMD-60, with the following specified parameters: temperatures in all zones of 125 °C, an adapter temperature of 140 °C, a molding head temperature of 145 °C in circumference, a screw rotation speed of 10 rpm, and an extruder main drive load of 80%, which is provided by the rotation of the raw material dispenser. The vacuum was not created in the degassing zone. After the extrudate exited the head, the board entered the calibrator and cooling bath, where its final molding took place at a water temperature of 14–16 °C. Then, the formed and cooled board entered the cutting device, where the boards were cut to a certain size. To improve the appearance and functional properties, the decking board was subjected to surface treatment by grinding and tufting. Samples for research were taken from the produced boards at the beginning, middle, and end of the batch.

The technological process of producing the WPC consisted of the following stages: preparation of components; drying and quality control of raw materials; preparation of a mechanical mixture in a paddle mixer (SKY WIN SWHL 600/1200) at a speed of 800 rpm; granulation of the resulting mass in a twin-screw extruder (sky win swmsz-3) at a temperature of 160 to 2000 °C; and extrusion of a decking board from which samples for research were cut.

**Figure 3:**
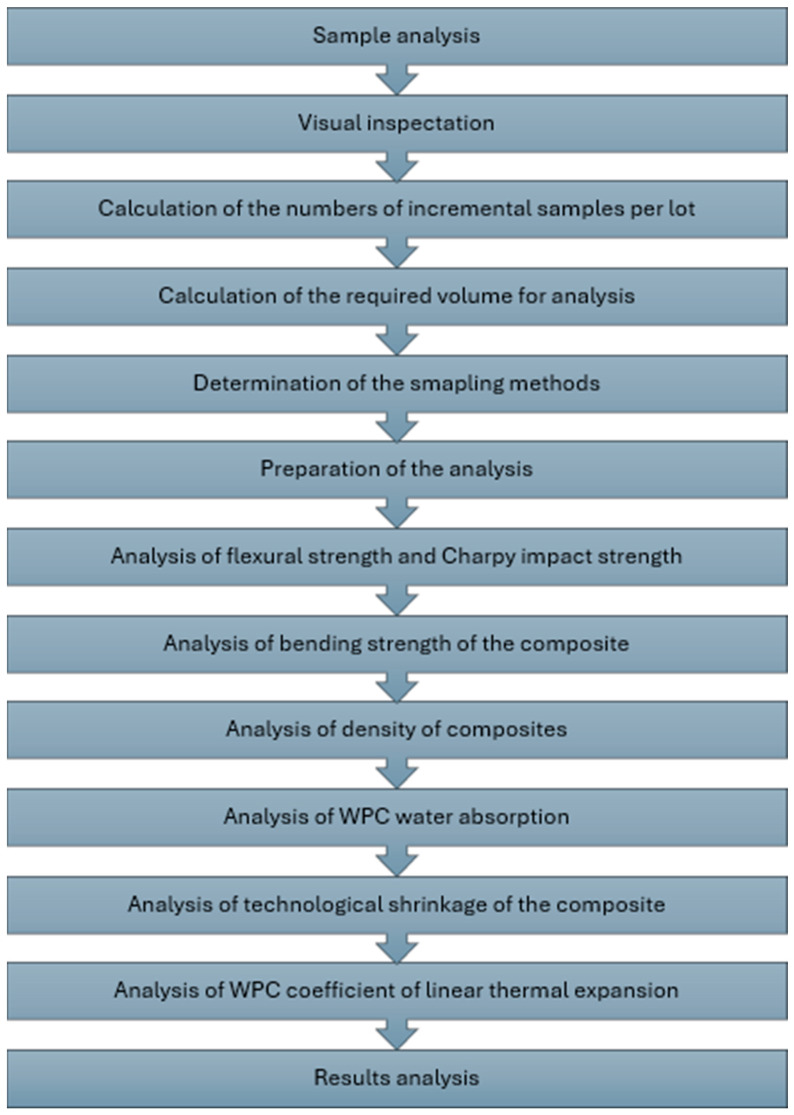
The scheme of the analysis and measurement process.

In the mechanical testing of samples, technological and operational properties were determined according to standard methods of research and testing of plastics. Statistical processing of the experimental data was performed by regression-correlation analysis. Measurements of the properties of each sample were repeated five times, with a confidence interval of 0.95. The scheme of the analysis and measurement process is depicted in Figure 3.

Experimental data were obtained according to existing standard methods and modern research methods:

- Charpy impact strength was determined according to ISO 179:2017, using notched specimens of 80 × 10 × 5 mm, with an impact energy of 2 J and a test temperature of 23 °C.
- Flexural strength was measured according to ISO 178:2019, using the three-point bending test, with specimens of 160 × 10 × 5 mm, a test speed of 2 mm/min, and a span length of 64 mm.
- Water absorption was determined according to ISO 62:2008, by immersing specimens in distilled water at 23 °C for 24 h, followed by weighing with an accuracy of 0.001 g.
- The coefficient of linear thermal expansion was measured according to ASTM D 696, using a dilatometer within a temperature range of −15 °C to 80 °C.
- Technological shrinkage was determined according to DIN 16901, by measuring the dimensional change in molded specimens in the transverse direction after 24 h of conditioning at 23 °C and 50% relative humidity.
- Density was determined according to ISO 1183-1, using the buoyancy method, with analytical balances and isopropyl alcohol as the immersion medium.

## 3. Results

**Figure 4:**
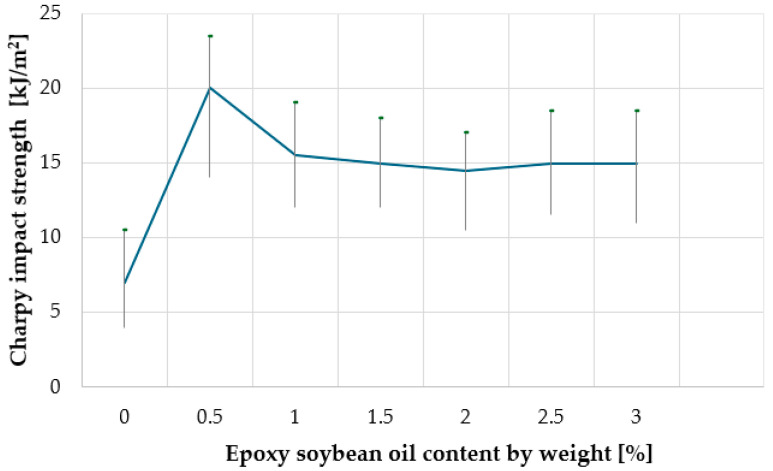
Dependence of the Charpy impact strength (on specimens with a cut) of the composite on the content of epoxy soybean oil.

**Figure 5:**
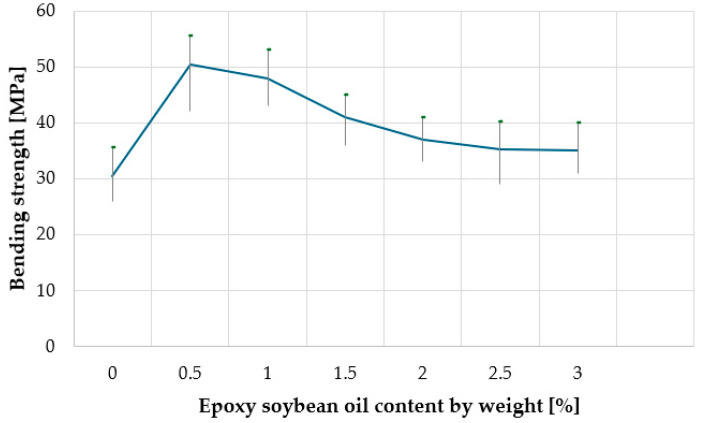
Dependence of the bending strength of the composite on the content of epoxy soybean oil.

**Figure 6:**
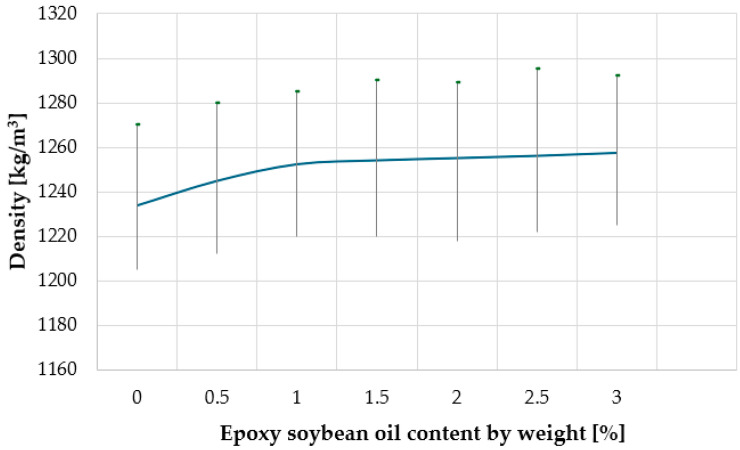
Dependence of the density of composites on the content of epoxidized soybean oil.

**Figure 7:**
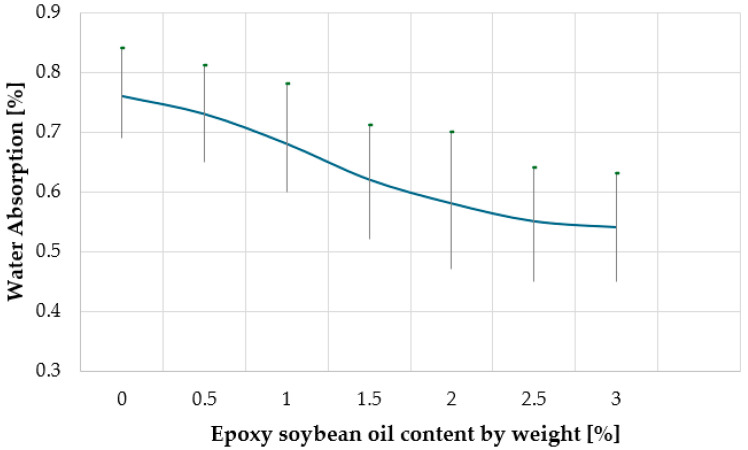
Dependence of WPC water absorption on the content of epoxidized soybean oil.

**Figure 8:**
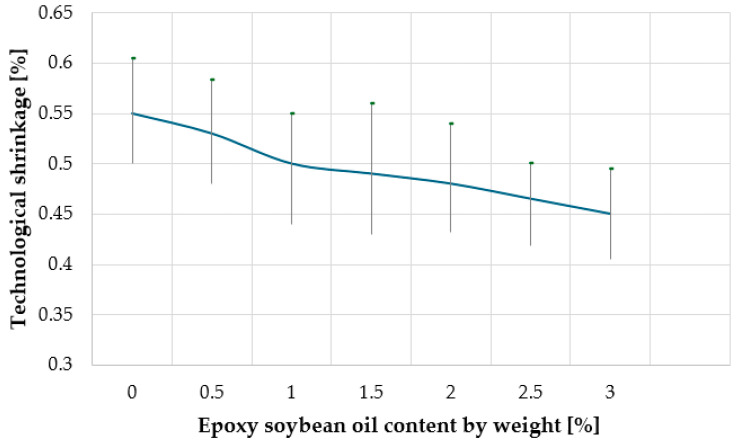
Dependence of the technological shrinkage of the composite on the content of epoxy soybean oil.

**Figure 9:**
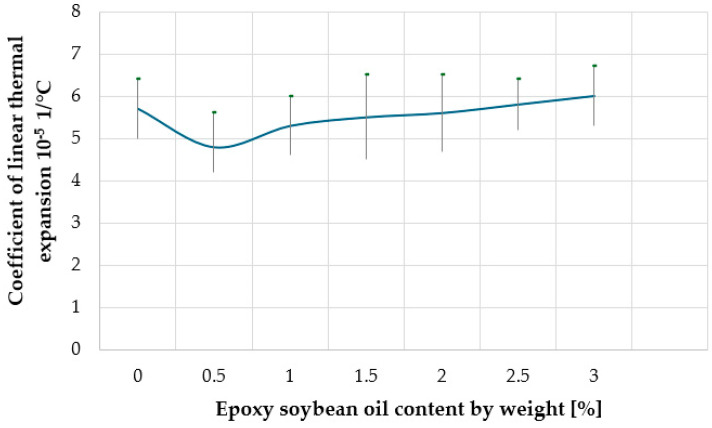
Dependence of the coefficient of linear thermal expansion of the composite on the content of epoxy soybean oil.

When determining the properties of a WPC material for outdoor use, physical and mechanical parameters are among the most informative. Previous studies have shown that for a WPC as a finishing material for facades and terraces, bending strength and Charpy impact strength are crucial. Figure 4 and Figure 5, respectively, show the effect of the content of epoxy soybean oil on the flexural strength and Charpy impact strength (on notched samples) of wood-polymer composites. Figure 4, Figure 5, Figure 6, Figure 7, Figure 8 and Figure 9 present the standard deviation of the measured values depending on the concentration of epoxy soybean oil.

Analyzing the data of Figure 4 and Figure 5, it can be stated that at the content of epoxidized soybean oil of 0.5 wt.% in the composition, the mechanical properties of the wood polymer composite improve: the Charpy impact strength (on notched samples) increases from 7.5 kJ/m^2^ to 20.0 kJ/m^2^ (almost three times), and the bending strength increases from 31.6 MPa to 50.8 MPa (by 60%). With a further increase in the content of epoxidized soybean oil to 3.0 wt.%, the mechanical properties of the composite deteriorate: the bending strength decreases to 34.2 MPa, and the Charpy impact strength (on notched samples) decreases to 15.2 kJ/m^2^. A statistical analysis was performed to assess the significance of these differences. The results indicate that the observed variations in impact strength and bending strength are statistically significant at the 0.95 confidence level, confirming the reliability of the trends presented in the study.

This strengthening mechanism can be explained by the fact that at low concentrations, epoxidized soybean oil acts as a plasticizer, improving the mobility of polymer chains and contributing to a more efficient distribution of stresses under load [18]. This leads to an increase in impact strength and flexural strength. However, when the concentration of epoxidized soybean oil increases to 1.5 wt.%, it can start to act as a defect, forming microscopic inclusions that become stress concentrators [19]. This leads to a decrease in flexural strength and impact strength, since the energy required to fracture the material is reduced due to premature crack formation in stress concentration areas. This effect is typical of excessive amounts of plasticizers in polymer composites [20].

In other words, this strengthening mechanism can be explained by the fact that the epoxy groups contained in epoxidized soybean oil interact with the hydroxyl groups of wood, and the nonpolar fatty acid moieties contained in soybean oil have good compatibility with polyethylene, thereby increasing the compatibility between the polymer and the wood. When the amount of epoxidized soybean oil in the composite increases, the polymer matrix plasticizes because the relatively small molecules of epoxidized soybean oil diffuse into the polymer, pushing out larger polyethylene molecules, surrounding them with a monolayer, and shielding the polar groups, resulting in a decrease in flexural strength and Charpy impact toughness.

This mechanism of reinforcement can be explained by the fact that the epoxy groups contained in epoxy soybean oil interact with the hydroxyl groups of wood, and the non-polar fatty acid fragments contained in soybean oil are compatible with polyethylene, and therefore, the compatibility between the polymer and wood increases. With an increase in the content of epoxy soybean oil in the composite, due to the fact that relatively small oil molecules diffuse into the polymer, push macromolecules apart, surround them with a monomolecular layer, and screen the polar groups, the polymer matrix plasticizes, and, as a result, the bending strength and Charpy impact strength decrease. Many manufacturers consider the density of composite building materials as a factor determining the weight of the profile (in terms of transportation costs), the ease of the installation of decks, and as a factor determining the consumption of raw materials. The density of the composite material also largely determines the service life and performance characteristics of products [18]. The decomposition of polymer and lignin from wood fibers during processing leads to the formation of volatile organic compounds, and thus to the porosity of the composite and a decrease in density. Figure 6 shows the dependence of WPC density on the content of epoxidized soybean oil.

As can be seen from Figure 6, with an increase in the content of epoxy soybean oil, the density of the composite increases from 1234 kg/m^3^ to 1258 kg/m^3^, indicating a more ordered structure. That is, epoxy soybean oil acts as a dispersant that prevents the agglomeration of wood particles during processing. The observed density increase aligns with the expected effect of ESO improving compatibility between the polymer and the wood, rather than a direct contribution from its own mass. Given that ESO has a density close to HDPE (~1000 kg/m^3^) and its evaporation is minimal at processing temperatures, its addition primarily influences particle distribution and matrix uniformity, rather than significantly altering the overall density.

Increased water absorption of WPC products can significantly reduce their durability, making them ineffective for exterior decoration. The main reasons for the increase in WPC water absorption are their porosity and low adhesion at the polymer–wood filler interface. The dependence of the water absorption of composites on the content of epoxied soybean oil is shown in Figure 7.

From the data in Figure 7, it can be concluded that with an increase in the content of epoxy soybean oil, the water absorption of wood-polymer composites decreases from 0.76% (in the absence of epoxy soybean oil in the composite) to 0.54% (with an epoxy soybean oil content of 3 wt.%). This positive trend can be explained, firstly, by the more ordered structure of the WPC (as indicated by the increase in density), and secondly, by the hydrophobic nature of epoxied soybean oil.

Technological shrinkage is a phenomenon that occurs during the production of products and is associated with their dimensional changes due to temperature, humidity, or other processing factors. In the case of wood-polymer composites, technological shrinkage can occur during the molding process or during the cooling of the material after production. shrinkage can affect the dimensional accuracy of the product and its appearance. In this work, we investigated the effect of the content of epoxy soybean oil on the technological shrinkage of the composite (Figure 8).

As can be seen from Figure 8, with an increase in the content of epoxidized soybean oil in the WPC to 3% by weight, the technological shrinkage decreases from 0.55% to 0.45%. This behavior is explained primarily by an increase in the density of the composite material, which leads to a decrease in the reorientation of polymer macromolecules during the cooling of the extrudate. Technological shrinkage was measured in the transverse direction to the extrusion flow. In WPC processing, shrinkage is typically anisotropic, with differences between the extrusion direction and the transverse direction. The alignment of polymer chains and wood fibers during extrusion often results in greater shrinkage along the extrusion direction, while transverse shrinkage is generally lower. In injection molding, shrinkage may be more uniform, but it depends on cooling conditions and pressure distribution. Lower technological shrinkage at a high ESO content has important implications for the production of large WPC profiles. Reduced shrinkage in the transverse direction helps minimize dimensional variations, making it easier to control the final shape and size of extruded products. Additionally, lower shrinkage reduces internal stresses, which can improve the long-term stability and durability of large WPC structures.

Thermal expansion and contraction is a universal phenomenon and is observed for all solids. The higher the ambient temperature, the higher the amplitude of vibrations of atoms and molecules of materials, which increases the effective volume of objects and, as a result, their size in all directions. The linear expansion–compression coefficient (k) can be calculated using the following formula: k = ΔL/(L × ΔT), where ΔL is the expansion or contraction of the material in the temperature range ΔT, and L is the initial size of the material at the beginning of the temperature range. The dependence of the linear coefficient of thermal expansion of the WPC on the content of epoxied soybean oil in the composite is shown in Figure 9.

Analyzing the data in Figure 9, it can be concluded that when 0.5% by weight of epoxy soybean oil is added to the composite, the coefficient of linear thermal expansion decreases by 15%. However, with a further increase in the content of epoxidized soybean oil in the composite, the coefficient of linear thermal expansion increases. This behavior can be explained by the fact that at low concentrations, ESO exhibits a weak plasticizing effect, while simultaneously improving the compatibility of wood particles with polyethylene and preventing their agglomeration. However, at higher concentrations, ESO begins to excessively plasticize the polymer matrix, increasing the mobility of macromolecules. This excessive plasticizing effect leads to structural weakening, as confirmed by the decline in mechanical properties at concentrations of 1.5 wt.% and above.

In order to verify the mechanism of interaction between the epoxy groups of epoxidized soybean oil and the hydroxyl groups of wood, IR spectra of epoxidized soybean oil, the WPC without epoxidized soybean oil, and the WPC with 0.5 wt.% epoxidized soybean oil were obtained (Figure 9) [26].

**Figure 10:**
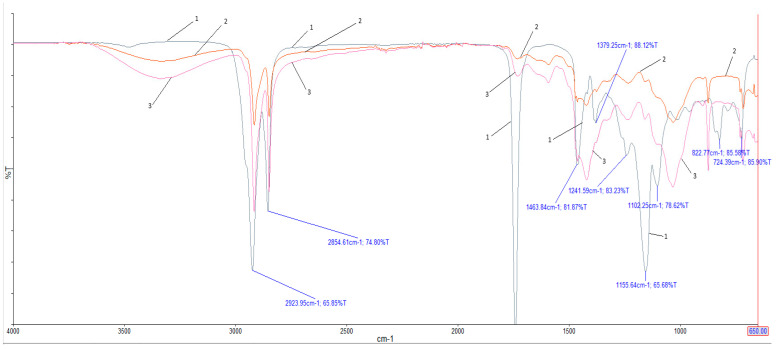
IR spectra of epoxidized soybean oil (1), WPC without epoxidized soybean oil (2), and WPC with epoxidized soybean oil content of 0.5 wt.% (3).

Analyzing the IR spectra (Figure 10), it is possible to see a shift in the peak at 1423.03 cm^−1^, which is in the spectrum of the pure composite, by 2.05 cm^−1^ in the long-wave direction, to 1420.98 cm^−1^, after the introduction of 0.5 wt.% epoxidized soybean oil. This band can be attributed to the deformation vibrations of the R-O-H group. Such a shift can be explained as a result of blocking interactions between hydroxyl groups of cellulose macromolecules in the surface layers of wood particles due to the reaction between epoxy and hydroxyl groups with the formation of ether alcohol fragments. At the temperatures used to process the composite mass into a board, this is entirely possible. In this way, the hydrophobic molecules of the epoxidized soybean oil chemically bond to the wood surface and improve its thermodynamic affinity with polyethylene [27,28].

## 4. Discussion

Their effectiveness as an alternative to traditional materials is well established. With the global trend toward using natural compounds, wood-polymer composites (WPCs) not only offer technological advantages over natural materials, but also consist primarily of renewable components [29].

However, most WPCs combine natural lignocellulosic fibers with plastics derived from fossil fuels, which raises concerns regarding the sustainability and carbon footprint. Additionally, the recycling of WPCs remains limited, as the extrusion process creates strong interfacial bonds, making it difficult to separate renewable and non-renewable components after processing.

Since terrace decking represents the largest share of WPC products, the incorporation of epoxidized soybean oil (ESO) as a finishing agent in composites based on recycled polymers and wood flour enables the production of cost-effective, high-quality decking boards with enhanced physical, mechanical, and operational properties. The use of recycled polymers in WPC formulations can significantly reduce environmental impact, lowering dependence on virgin fossil-based plastics (Table 3). Further research into biodegradable polymer matrices and advanced recycling techniques could improve the sustainability of WPCs, addressing these concerns.

Thus, wood-polymer composite flooring boards have a number of advantages over natural wood boards, which makes them suitable for use in the construction and decoration of exterior structures. The results of many studies show the effect of using a WPC or a combination of WPC/wood compared to classic materials [30,31]. However, it should be noted that WPC decking boards are generally more expensive than pressure-treated pine boards, with prices sometimes twice as high. Despite the higher initial cost, WPC offers greater durability, resistance to moisture, insects, and decay, and requires minimal maintenance, unlike pine, which may need regular sealing, staining, or replacement due to weathering.

## 5. Conclusions

According to the results of the studies on the effect of epoxidized soybean oil on the properties of a WPC based on high-density recycled polyethylene, it was found that the use of epoxy soybean oil in wood-polymer composites reduces technological shrinkage and water absorption, which makes it possible to use these composites to produce products with more accurate dimensions that operate in high-humidity conditions.

The rational content of epoxidized soybean oil, which provides the highest mechanical and operational performance in the WPC, is 0.5 wt.%, but with a further increase in the content of epoxidized soybean oil, its physical, mechanical and operational properties deteriorate.

Since soybean oil is a plant-based product, its use in wood-polymer composites has a positive environmental impact and allows for the production of WPC boards at a lower cost compared to conventional WPC formulations. This cost reduction is achieved through improved processing efficiency and reduced reliance on synthetic additives, making an ESO-modified WPC a more economical alternative within the WPC market.

The ESO-modified composites could be processed at lower temperatures, which can reduce energy consumption and even mitigate equipment wear over time. These advantages highlight the eco-friendly nature of ESO and its potential to lower manufacturing costs while maintaining or improving material performance. The results have important implications for industrial applications of WPCs made with recycled polymers. Improved dimensional stability means that extruded or molded WPC products will retain their intended shape and dimensions more reliably, easing installation and improving fit in construction uses. The reduction in water absorption observed at optimal ESO levels translates to greater durability in outdoor or humid environments, as the composites will be less prone to moisture-induced swelling, decay, or freeze–thaw damage over time. Furthermore, the enhanced thermal stability and lower melting temperature of ESO-containing composites suggest easier processing and the possibility of faster production cycles or the use of lower processing temperatures in existing industrial equipment. Together, these improvements can broaden the range of applications for WPC materials—for example, in decking, cladding, or outdoor furniture—by improving their service life and reliability. Properties such as moisture resistance and dimensional stability were measured in accelerated tests, but real-world factors like prolonged UV exposure, seasonal temperature fluctuations, and biological attack (mold or microbes) were not fully simulated. Future research should address the above limitations to fully realize the potential of ESO in wood-polymer composites. Long-term performance testing is especially critical: extended outdoor exposure trials in diverse climates would clarify how ESO-modified WPCs endure years of weathering, including UV radiation, rain/humidity, and temperature cycling. Such studies will determine if the initial improvements in water resistance and dimensional stability are retained over the service life, or if issues like plasticizer leaching or the biodegradation of the ESO emerge after prolonged exposure.

## Figures and Tables

**Table 1:** Physical and mechanical properties of recycled high-density polyethylene.

Indicator	Value
Appearance	Dark green granules 3–5 mm in size
Density, kg/m^3^	956
Vick softening point, °C	130
Melt flow index, g/10 min	1.0
Curl flow, mm	370
Flexural strength, MPa	50
Charpy impact strength (on unnotched samples), kJ/m^2^	samples do not collapse
Tensile yield strength, MPa	22.3
Elongation at break, %	570

**Table 2:** Quality characteristics of wood flour grade M200.

Name of the Indicator	Value
Moisture, %, not more	5
Mass fraction of ash, %, not more	1.0
Mass fraction of colored impurities, %, not more	4.0
Mass fraction of metallomagnetic impurities, %, not more	0.0016
Bulk density, kg/m^3^	100 to 140
Mass fraction of residue on the grid 0.18, %, not more	5.0
Mass fraction of residue on the grid 0.25, %, not more	0.4
Mass fraction of resins and oils, %, not more	5.0

**Table 3:** Comparative characteristics of pine and WPC.

Parameters	WPC	Pine
Appearance	Smooth, no knots	Rough, knots present
Susceptibility to rot and mold	Not susceptible	Susceptible
Susceptibility to cracking	Not susceptible	Prone
Need for special care	Does not require	Requires
Resistance to UV radiation	Resistant	Not resistant
Bending strength, MPa	50.8	49.5
Water absorption, %	0.72	185
Charpy impact strength, kJ/m^2^	20.0	35.0
Coefficient of linear thermal expansion, 1/°C	4.8 × 10^−5^	29 × 10^−6^
Average service life, years	20–50	10–20

## Data Availability

The original contributions presented in this study are included in the article. Further inquiries can be directed to the corresponding author.
